# Data on inhibitive performance of chloraphenicol drug on A315 mild steel in acidic medium

**DOI:** 10.1016/j.dib.2018.05.108

**Published:** 2018-05-23

**Authors:** A.A. Ayoola, O.S.I. Fayomi, S.O. Ogunkanmbi

**Affiliations:** a Chemical Engineering Department, Covenant University, Ota, Nigeria; b Mechanical Engineering Department, Covenant University, Ota, Nigeria; c Surface Engineering Research Centre, Tshwane University of Technology, South Africa

**Keywords:** Acidic medium, Corrosion, Inhibitor, Mild steel

## Abstract

The inhibitive characteristics of A315 mild steel in 0.1 M solution of Hydrochloric Acid with varied concentrations of the inhibitor (chloramphenicol drug) was studied using weight loss (gravimetry) technique, open circuit potential (OCP) and linear polarization method. The experimental data obtained from the methods used shows that an increase in inhibition efficiency of the inhibitor is characterized by a decrease in corrosion rate. Hence, chloramphenicol drug is an efficient corrosion inhibitor for Mild Steel in Hydrochloric acid medium.

**Specifications Table**

**Table:** 

Subject area	*Materials Science Engineering*
More specific subject area	*Corrosion Engineering*
Type of data	*Table, image*
How data was acquired	The inhibitive characteristics of A315 mild steel in 0.1 M solution of Hydrochloric Acid with varied concentrations of the inhibitor (chloramphenicol drug) was studied using weight loss (gravimetry) technique, open circuit potential (OCP) and linear polarization method. Autolab potentiostat galvanostat equipment (PGSTAT101) was used for the electrochemical analysis, evaluation of corrosion inhibitor, study of reaction mechanism of anodic oxidation and corrosion study of mild steel in the acidic media. Spark Atomic Emission Spectrometer was used for the classification of the ferrous material used. NOVA 2.1 software was used for the electrochemical study.
Data format	Raw, Analyzed
Experimental factors	The concentration of chloramphenicol drug (as corrosion inhibitor) was varied during the electrochemical process. The mild steel coupons were abraded with emery papers of three grades (P609, P1000C and P1200A) and the surface became very smooth and silver coloured. To remove any surface impurities that arose from cutting and polishing of the mild steel coupons, distilled water was used to wash the cut squares thoroughly. And acetone was used to rinse the cut squares which were then dried at ambient temperature before been stored in a desiccator before usage.
Experimental features	Mild steel coupons after pretreatment were used for the electrochemical experiment. The electrochemical process was performed using Autolab potentiostat galvanostat equipment (PGSTAT 101), NOVA 2.1 software and a 3-electrode cell containing 100 ml of 0.1 M Hydrochloric acid (with and without inhibitor) at 30 °C. A graphite rod was used as the auxiliary electrode, silver chloride electrode (Ag/AgCl) was used as the reference electrode and the mild steel coupon was connected to a specimen holder to serve as the working electrode.
	Potentiodynamic study was carried out by considering −1.5 to +1.5 voltage range at a scan rate of 0.005 V/s. Linear sweep voltammetry (LSV) staircase was conducted and corrosion current was measured for each of the experimental runs. The Tafel plots of potential E(V) against log current (I) were generated to obtain corrosion potential (Ecorr) and corrosion current density (jcorr). Also, the corrosion rate and inhibition efficiency were evaluated using NOVA 2.1 software.
Data source location	Department of Chemical Engineering, Covenant University, Ota and Mechanical Engineering Department, Covenant University, Ota, Nigeria.
Data accessibility	Data are available within this article

**Value of the data**

• The given data will enable authors in Corrosion Engineering profession the inhibitive behavior of chloramphenicol in acidic medium.
• The data can be used to examine the relationship between the process variables (as such inhibitor concentration, exposure time) and inhibition efficiency.
• The data could be used to obtain the inhibition efficiency of chloramphenicol (as inhibitor) at any given inhibitor concentration.
• The data acquired revealed that Langmuir adsorption model was the most suitable adsorption model.

## 1 Data

**Fig. 1:**
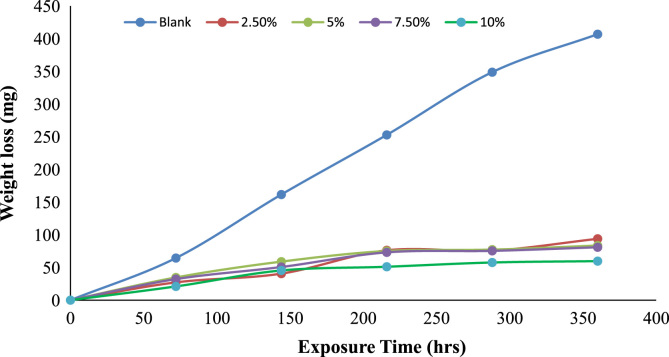
Trend of weight loss with exposure time during electrochemical process.

**Fig. 2:**
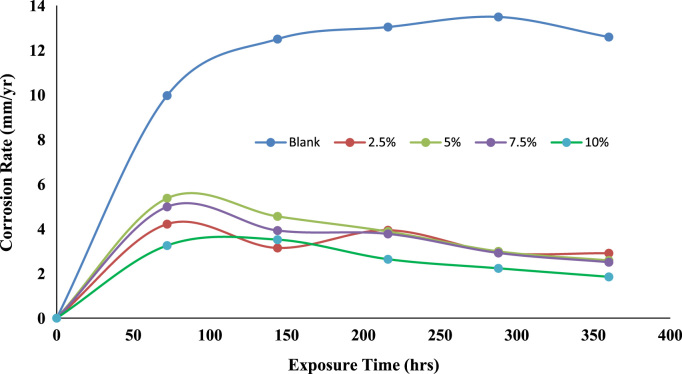
Trend of corrosion rate with exposure time.

**Fig. 3:**
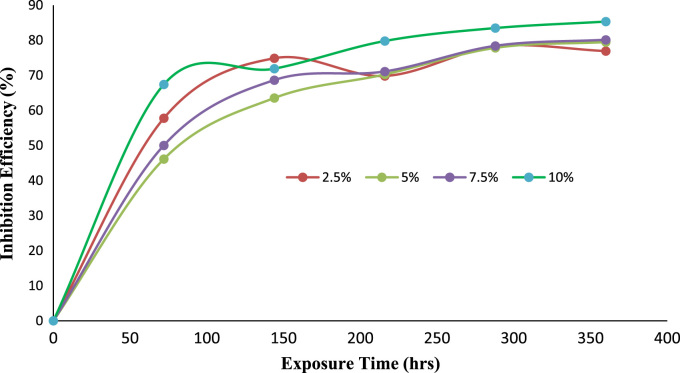
Trend of inhibition efficiency with exposure time.

**Fig. 4:**
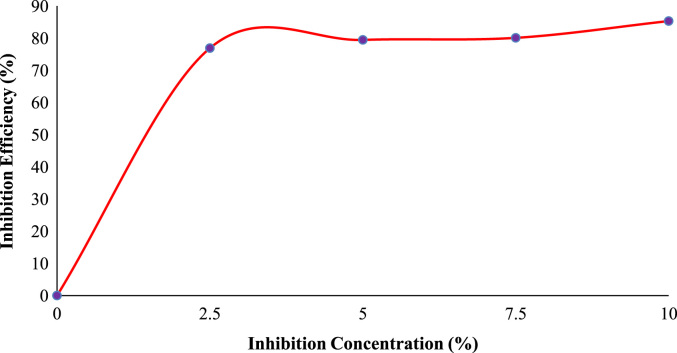
Trend of inhibition efficiency with inhibitor concentration at 360 hours.

**Fig. 5:**
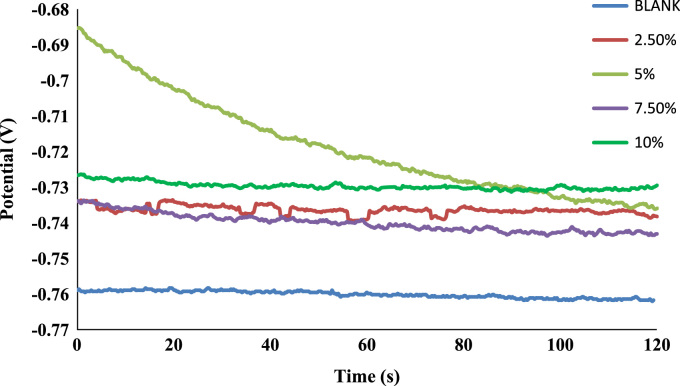
OCP values of the uninhibited and inhibited samples.

**Fig. 6:**
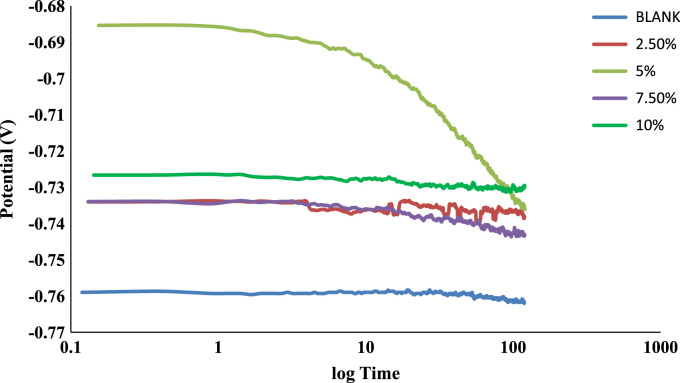
OCP values versus log time of the uninhibited and inhibited samples.

**Fig. 7:**
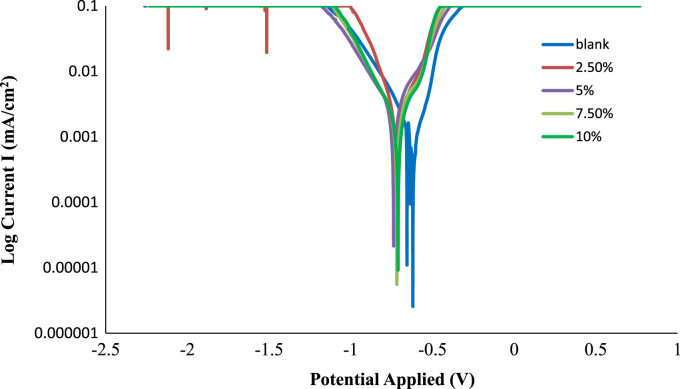
Linear polarization plots for mild steel in 0.1 M HCl with and without chloramphenicol inhibitor at 30 °C.

**Fig. 8:**
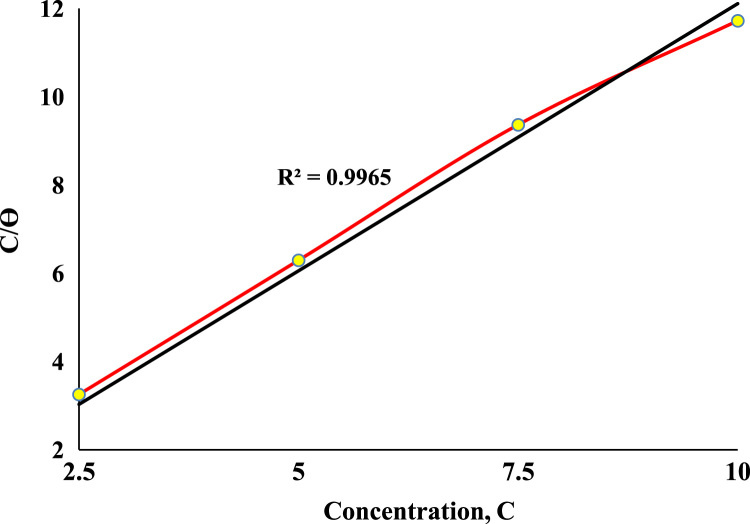
Langmuir Adsorption plot for chloramphenicol adsorption process.

The weight loss, corrosion rate inhibition efficiency, surface coverage and OCP values of the uninhibited and inhibited samples were determined during the electrochemical process using chloramphenicol as the corrosion inhibitor. These data are presented in Table 1 and Fig. 1, Fig. 2, Fig. 3, Fig. 4, Fig. 5, Fig. 6, Fig. 7, Fig. 8.

**Table 1:** Gravimetry data for mild steel in 0.1 M HCl in the presence of inhibitor concentrations (chloramphenicol) at 360 h.

**Inhibitor Conc. (%)**	**Weight loss (mg)**	**Corrosion potential (Ecorr, V)**	**Current density (jcorr, A/cm**^**2**^**)**	**Corrosion rate (mm/year)**	**Inhibition efficiency (%)**	**Surface coverage (ϴ)**
0	406.7	−0.656	0.0009	12.591	0	0
2.5	93.9	−0.717	0.0003	2.907	76.912	0.769
5.0	83.5	−0.737	0.0002	2.585	79.469	0.795
7.5	80.9	−0.718	0.0002	2.505	80.108	0.801
10	59.7	−0.710	0.0001	1.848	85.321	0.853

## 2 Materials and methods

Mild steel coupons after pretreatment were used for the electrochemical experiment. The electrochemical process was performed using Autolab potentiostat galvanostat equipment (PGSTAT 101), NOVA 2.1 software and a 3-electrode cell containing 100 ml of 0.1 M Hydrochloric acid (with and without inhibitor) at 30 °C. A graphite rod was used as the auxiliary electrode, silver chloride electrode (Ag/AgCl) was used as the reference electrode and the mild steel coupon was connected to a specimen holder to serve as the working electrode, as carried out in previous work [1], [2].

The bath preparation for electrochemical (corrosion) process was designed with varied concentration of chloramphenicol as corrosion inhibitor (2.5–10%). Total time duration of 360 h was considered for the experiment, but with intermittent weighing of the mild steel in every 72 h [1], [2], [3], [4].

Potentiodynamic study was carried out by considering −1.5 to 1.5 voltage range at a scan rate of 0.005 V/s. Linear sweep voltammetry (LSV) staircase was conducted and corrosion current was measured for each of the experimental runs. The Tafel plots of potential E(V) against log current (I) were generated to obtain corrosion potential (Ecorr) and corrosion current density (jcorr). Also, the corrosion rate and inhibition efficiency were evaluated using NOVA 2.1 software.
